# Evaluating the Long-Term Efficacy of Four Active Ingredients Against *Rhyzopertha dominica* (F.) (Coleoptera: Bostrichidae) and *Sitophilus oryzae* (L.) (Coleoptera: Curculionidae) on Stored Sorghum in the United States

**DOI:** 10.3390/insects17030273

**Published:** 2026-03-03

**Authors:** Tanner Liba, Kun Yan Zhu, Deanna S. Scheff

**Affiliations:** 1 Department of Entomology, Kansas State University, Manhattan, KS 66506, USA; tliba@ksu.edu (T.L.); kzhu@ksu.edu (K.Y.Z.); 2 Center for Grain and Animal Health Research, Agricultural Research Service, United States Department of Agriculture, 1515 College Avenue, Manhattan, KS 66502, USA

**Keywords:** integrated pest management, grain protectants, residual efficacy, bulk storage, methoprene, pyrethrum, pyrethroid, spinosad, post-harvest storage, stored-product pests

## Abstract

Grain protectants are insecticide formulations applied directly to raw grain to prevent and control insect infestations in bulk storage; however, their efficacy depends on insect species, commodity, and grain quality. The objective of this study was to determine the residual efficacy of four commercially available grain protectants, Gravista^®^ (deltamethrin + methoprene + PBO), Diacon^®^ IGR (methoprene), Sensat^TM^ (spinosad), and EverGreen^®^ (pyrethrin), applied to sorghum and held for 28 weeks, against *Rhyzopertha dominica* (F.) and *Sitophilus oryzae* (L.). Subsamples were collected every four weeks and infested with 10 adults of either species. Adult mortality was assessed after seven days and progeny, frass, and insect-damaged kernels were evaluated after eight weeks. *Rhyzopertha dominica* was more susceptible to all insecticides compared to *S. oryzae*, and had fewer progeny compared to control sorghum. Spinosad-treated sorghum resulted in 100% adult mortality in *R. dominica* compared to 30–39% in *S. oryzae.* There was an overall decline in kernel moisture content, which may have impacted progeny of both species in the control and insecticide-treated sorghum. These findings highlight the influence of insecticide formulation, insect species, and grain moisture content on the long-term efficacy of grain protectants applied to sorghum.

## Simple Summary

Stored product pests such as the lesser grain borer and the rice weevil cause significant damage to grain products during post-harvest storage. In the United States, grain protectants are commonly applied to grain prior to storage to prevent infestation, but their long-term performance depends on environmental conditions inside the storage structure and the target pest. In this study, we evaluated the residual effectiveness of four commercially available grain protectants applied to sorghum stored for 28 weeks in a grain bin. We evaluated adult mortality, progeny production, frass weight, and the percentage of insect-damaged kernels every four weeks. The lesser grain borer adults were more susceptible to each treatment compared to rice weevils, and the insecticide that contained the active ingredient spinosad was the most effective against both species. In addition, the overall moisture content of the sorghum declined over the course of experiment, resulting in the reduction in progeny of both species in both the control and insecticide-treated sorghum. Overall, these findings highlight the importance of kernel moisture content and the varying efficacy of grain protectants in reducing pest damage from different species of stored product pests.

## 1. Introduction

Stored product pests are a group of arthropods that infest and consume stored products, posing a significant threat to global food security. These pests, which can be found in storage facilities, shipping containers, and commercial markets, target both intact and broken grain kernels within storage facilities and across supply chains [1]. In the United States (U.S.), damage caused by infestations contributes to an estimated $2.5 billion in annual economic losses due to product degradation, damage, and contamination [2]. Integrated pest management (IPM) is a comprehensive approach to prevent, control, and manage product loss due to infestation by combining preventative and corrective measures to safeguard stored grain supplies [3]. One key method of pest control for stored bulk grain is the application of grain protectants [4]. Grain protectants are insecticide formulations that are applied directly to freshly harvested grains as they are loaded into storage bins or silos [3,4]. For maximum efficacy, insects must come into direct contact with the insecticide either through ingestion of treated grain or through contact on the exoskeleton when the insect is traversing treated grain. A wide range of commercially available protectants exists in the U.S., each with their own distinct mode of action determined by the active ingredients (a.i.) in the insecticide formulations. Some formulations target all stages of a pest’s lifecycle, while other formulations incorporate an insect growth regulator (IGR) that disrupts normal juvenile development of the insect and inhibits development to adulthood [5]. There are a number of factors to consider before treatment to ensure maximum efficacy and longevity of grain protectants. These factors include the biological and behavioral difference between pest species of concern [6,7] the type of commodity treated [7,8], and abiotic storage parameters such as temperature and humidity [9,10].

*Sorghum bicolor* (L. Moench), widely referred to as sorghum, great millet, or milo, ranks among the five most significant cereal crops worldwide [11]. In 2023, global sorghum production reached 118.9 million tons, with the U.S. ranking among the top producers at 20.46 million tons [12]. Like other stored commodities, sorghum is susceptible to a range of pest species that cause damage during long-term periods of storage post-harvest. Two species of particular concern for stored grain are *Rhyzopertha dominica* (F.)*,* the lesser grain borer, and *Sitophilus oryzae* (L.), the rice weevil. These insect pests damage grain by internally developing within the kernel as larvae and emerging as adults, leaving behind hollowed grains and producing substantial frass and other contaminants that further degrade grain quality [13,14]. While these two species occupy the same food source, their behavior and oviposition strategies differ markedly as *R. dominica* adults deposit eggs on the surface of kernels, whereas *S. oryzae* females bore into the kernel prior to oviposition, placing eggs within the grain [15].

Despite its widespread cultivation and economic significance worldwide, research on the long-term effectiveness of grain protectants on sorghum is understudied, compared to other staple cereal grains like wheat, rice, and maize. While studies on these grains provide a useful framework for understanding how protectants might behave on sorghum, key differences in grain structure, chemical composition, and storage conditions mean that direct studies on sorghum are necessary to develop optimized treatment strategies. Without targeted research, assumptions based on other grains may not fully capture the unique challenges associated with protecting stored sorghum from infestation and contamination. Given the growing demand for sorghum as both a food and industrial crop [16], further investigation into its susceptibility to stored product pests and the long-term efficacy of available insecticidal treatments is essential to ensure stored product security. Therefore, the objective of this study was to evaluate the residual efficacy of commercially available U.S. grain protectants, Gravista^®^, Diacon^®^ IGR, EverGreen^®^, and Sensat^TM^, applied to sorghum against *R. dominica* and *S. oryze*, two species commonly associated with stored grain [15].

## 2. Materials and Methods

### 2.1. Insects and Commodity

The insects used in this study were pesticide susceptible laboratory strains of *R. dominica* and *S. oryzae* from colonies previously maintained on hard red winter wheat at the USDA–ARS, Center for Grain and Animal Health Research (CGAHR) in Manhattan, Kansas for more than 30 years. Prior to this experiment, *R. dominica* and *S. oryzae* were reared on commercial sorghum (Cargill^®^, Salina, KS, USA) in an environmental chamber set at 27 °C and 60% relative humidity, in continual darkness (Percival, Perry, IA, USA), based on published rearing procedures for both species [17]. Both species were reared on sorghum for at least three generations prior to their use in any experiment.

Rearing procedures on sorghum was as follows. Approximately 100 adult *R. dominica* or *S. oryzae* were added to 400 g of sorghum and held at 27 °C and 60% relative humidity, in continual darkness, for seven days. After seven days, the adults were removed, and the sorghum returned to the environmental chamber until adult progeny were observed, approximately four–five weeks post-parental adult removal. For this experiment, two-week-old mixed sex adult *R. dominica* and *S. oryzae* were used.

The sorghum used in this experiment was the same commercial sorghum used for colony maintenance and was first frozen for a minimum of 48 h to kill any insects that may be present in the grain. The sorghum was sieved using a Carter Day Dockage Tester and a #6 (5/64″, 1981 µm) sieve and a #1 (2.5/64″, 990 µm) sieve (Carter Day International, Inc., Minneapolis, MN, USA). The sorghum was then processed through a thresher machine (Precision Machine Co. Inc., Lincoln, NE, USA) to remove any glumes, dust, and any other foreign material.

### 2.2. Insecticide Formulations and Commodity Treatments

Four different commercially available grain protectant formulations were used in this study, with water serving as the control. The first contained the active ingredient methoprene, with the tradename Diacon^®^ IGR (Wellmark International, Schaumburg, IL, USA). The formulation consists of 33.6% a.i. methoprene or 288 g/L a.i and is henceforth referred to as Diacon^®^ IGR. Diacon^®^ IGR was applied at the label rate of 210 mL insecticide per 18.9 L water for 1000 bushels of sorghum. To enable treatment at this rate, 0.6 mL of Diacon^®^ IGR was mixed with 50 mL water and 1.9 mL of insecticide was applied to treat 2.5 kg of sorghum.

The second insecticide used in this study was a 5% a.i. pyrethrin concentrate with the tradename EverGreen^®^ (McLaughlin, Gormley & King, Minneapolis, MN, USA). The exact concentration of the pyrethrin composition cannot be disclosed due to trade secrets. EverGreen^®^ was applied at the label rate of 177.44 mL per 3.7 L of water for 1000 bushels of sorghum. To treat at this rate, 2.4 mL of EverGreen^®^ was mixed with 50 mL of water and applied at the rate of 1.9 mL to treat 2.5 kg of sorghum.

The third grain protectant used in this study consisted of a liquid combination of deltamethrin + methoprene + piperonyl butoxide (PBO), with the tradename of Gravista^®^ (Central Garden and Pet, Schamburg, IL, USA), and is henceforth referred to by the trade name Gravista^®^. The Gravista^®^ consists of 1.20% a.i. deltamethrin, 2.85% a.i. methoprene, and 33.30% a.i. (PBO), or 45 g of deltamethrin, 103.9 methoprene, and 1211.1 g of PBO in 3.8 L of water. Gravista^®^ was applied at the label rate of 937 mL insecticide per 18.9 L water for 1000 bushels of sorghum. To enable treatment at this rate, 2.5 mL of Gravista^®^ was mixed with 50 mL of water and 1.9 mL of insecticide was applied to treat 2.5 kg of sorghum.

The fourth grain protectant to be used contained the active ingredient spinosad, with the tradename Sensat^TM^ (Bayer CropScience, Research Triangle Park, NC, USA), and this will henceforth be referred to the tradename Sensat^TM^. The formulation for Sensat^TM^ consists of 8.66% (concentration by weight) a.i. spinosad. Sensat was applied at the label rate of 290 mL insecticide per 18.9 L water for 1000 bushels of sorghum. To enable treatment at this rate 0.8 mL of Sensat was mixed with 50 mL water and 1.9 mL of insecticide was applied to treat 2.5 kg of sorghum.

Three replicate insecticide formulations were prepared for each treatment, and one formulation was used per sorghum treatment (bin), and thus this represents three independent replications per insecticide formulation. The insecticide applications were made using an artist spray brush (Model 100^TM^, Badger, Franklin Park, IL, USA), with only water being applied as the control. The insecticide or water was sprayed on the sorghum as the grain was gently poured into a 0.29 × 0.29 × 0.15 m (L × W × H), 15-L plastic bin (Sterlite^®^, Townsend, TN, USA) from a 0.95 L glass jar. After the insecticide application, each bin containing treated sorghum was manually shaken for 20 s to ensure an even coating of the insecticide. This methodology was designed to mimic the application of a grain protectant being applied during the grain bin loading process. The bins were fitted with a vented lid and allowed to dry for 24 h under ambient conditions. This application process was replicated three times for each insecticide formulation and species combination. The treatment applications for *R. dominica* and *S. oryzae* were conducted in separate weeks.

### 2.3. Efficacy Bioassays of Grain Protectants

Approximately 24 h after insecticide applications, the treated sorghum was transferred to an 18.9 L plastic bucket fitted with two screened holes on the lid to allow for air movement during storage. Prior to closing the lids, five 50 g subsamples were taken from each of the three buckets that were prepared for each insecticide treatment (*N* = 15) and the subsamples were placed in a 0.18 L plastic bottle with a vented lid for a total of 15 bottles per insecticide and insect species combination (week 0). The buckets with the remaining sorghum were placed on the bottom of an empty 112-ton metal grain bin located at the USDA-ARS-CGAHR campus. There was no artificial light source in the grain bins, and the only light source was from small openings, cracks, or vents in the grain bin. Separate empty grain bins were used to store sorghum for the *R. dominica* and *S. oryzae* experiments.

Five 50 g subsamples were subsequently taken from each bucket every four weeks during the 28-week storage period. In each of the untreated sorghum buckets, one HOBO temperature/humidity data logger (Onset, Bourne, MA, USA) was placed inside to monitor the environmental conditions inside the bucket during the 28-week storage period, with temperature and relative humidity being logged every eight hours each day (*N* = 3).

**Figure 1:**
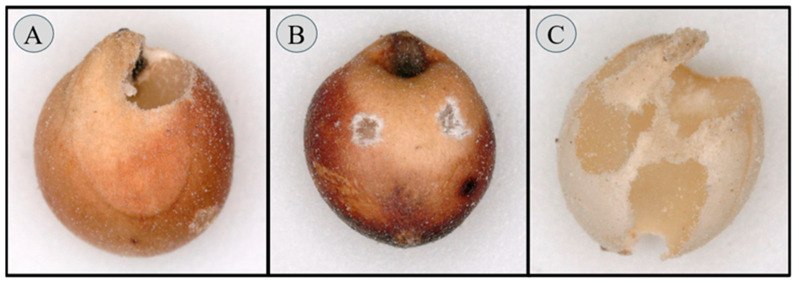
Sorghum kernels exhibiting (**A**) adult insect emergence hole, (**B**) adult feeding on the outside of the kernel, and (**C**) extensive damage caused by insects including adult feeding and emergence holes.

For each insect species being tested, bottles containing the individual 50 g subsamples were taken into the lab and 10 two-week-old mixed-sex adults of *R. dominica* or *S. oryzae* were added to an individual bottle and held for seven days in an environmental chamber set at 27 °C and 65% r.h. in complete darkness. After seven days, adult *R. dominica* or *S. oryzae* were removed using a 600 µm opening sieve and a catch pan (Dual Manufacturing Co., Chicago, IL, USA), and adults were assessed for mortality using a digital microscope (Dino-Lite 3.0 Edge Digital Microscope, Dunwell Tech, Inc., Torrance, CA, USA). Dead adults were those that did not move when gently prodded with forceps. The bottles were returned to the environmental chamber and held for an additional eight weeks to allow for adult progeny emergence. After eight weeks, bottles were frozen for 48 h and then the sorghum was sifted through a 1700 µm opening, 600 µm opening, and a catch pan sieve stack. Adult beetles were retained on the top of the 1700 µm sieve, the sorghum kernels were retained on the 600 µm sieve, and frass was collected in the catch pan. The number of adult progeny and weight (g) of the frass was recorded. After the sorghum samples were sieved, three subsamples of approximately five grams were randomly taken from each bottle and analyzed under a stereomicroscope to observe any insect-damaged kernels (IDKs). The IDKs consisted of kernels that exhibited adult emergence holes (Figure 1A), signs of adult feeding (Figure 1B) and/or extensive feeding damage (Figure 1C). The total percentage of IDKs from each bottle was calculated using Equation (1).

(1) $$Percent\;IDKs=[\frac{No.of\;Insect\;Damaged\;Kernels}{No.of\;Undamaged\;Kernels+No.of\;Insect\;Damaged\;Kernels}]\times 100$$

The sorghum moisture content was also measured every four weeks using a grain moisture analyzer (Mini Gac^®^ 2500, DICKEY-john^®^, Auburn, IL, USA). A sample of sorghum was taken from each control bucket (*N* = 3), and the mean moisture content among the three samples was determined.

### 2.4. Data Analysis

The mean daily temperature (°C) and % r.h. recorded by the three HOBO data loggers were combined and averaged per week for the study. The mean and standard error (±SE) for each storage week were calculated and reported. Data on the temperature and r.h. was subjected to a one-way analysis of variance (ANOVA), with the main factor of storage time using Statistical Analysis Software (SAS Version 9.4, SAS Institute, Cary, NC, USA). The mean (±SE) sorghum moisture content was determined and tested for normality via the proc univariate function in SAS (SAS Version 9.4, SAS Institute, Cary, NC, USA). Moisture content data for each time point was then compared using a one-way ANOVA for the main factor of storage time.

Data analysis was initially performed on each insect species separately to test for the main effects of insecticide treatment, storage time, and their interaction. Due to the lack of variation for some insecticides over time, we subsequently analyzed each combination of insecticide and insect species separately with time as the main effect with a total of *N* = 15 replicates per each timepoint. The mean (±SE) for the number of adult progeny and frass was first tested for normality and then transformed to log_10_ (x + 1) scale prior to statistical analysis [18]. The adult mortality and percentage IDKs were transformed into angular values prior to statistical analysis after they were found to be non-normal [18]. Data on the percentage of adult mortality, progeny, frass, and percentage IDKs were subjected to a two-way ANOVA, with the main factors of insecticide treatment and storage time as the main factors. If the ANOVA was significant (*p* < 0.05), differences among the treatments were determined by separating means using LS Means with Tukey’s Honestly Significant Difference (HSD) test (*p* < 0.05).

## 3. Results

### 3.1. Seasonal Changes in Temperature, Relative Humidity, and Moisture Content

**Figure 2:**
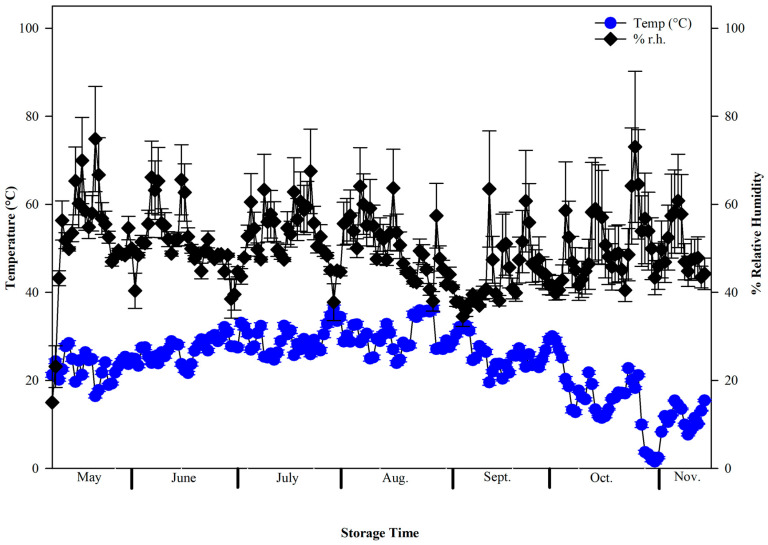
The mean (±SE) daily temperature (Temp, °C) and percent relative humidity (% r.h.) during the experimental period from May 2023 to November 2023.

The main effect of storage time (weeks) was significant for the mean weekly temperature (*F* = 141.86; df = 27,560; *p* < 0.05) and relative humidity (*F* = 7.32; df = 27,560; *p* < 0.05). The mean weekly temperature increased from ~25 °C in week 1 to a maximum daily temperature of ~36 °C at the 12-week period (Figure 2). This period equates to July, which is during the summer months in the U.S. and typically the warmest period in Kansas. Following this peak, the average daily temperatures began to decline until the completion of the study. The cooler temperatures observed during 22–28 weeks of storage (October–mid-November), corresponded to autumn weather in Kansas. In general, sorghum is harvested in September–October in Kansas and would be associated with environmental conditions associated with storage periods >18 weeks in this study (Figure 2). The mean daily relative humidity ranged from 23 to 75% throughout the storage time (Figure 2). 

The effect of storage time on the grain moisture content was significant (*F* = 40.39; df = 7, 16; *p* < 0.0001) (Table 1). The initial moisture content of the sorghum was 13.3% and consistently dropped throughout the duration of the study and was 9.9% after 28 weeks.

### 3.2. Effect on Adult Mortality

#### 3.2.1. *Rhyzopertha dominica*

The main effects of insecticide treatment (*F* = 78.60; df = 4595; *p* < 0.001) and the interaction of treatment × storage time (*F* = 20.31; df = 39,560; *p* < 0.001) were significant for *R. dominica* adult mortality. However, the main effect of storage time was not significant (*F* = 0.87; df = 7592; *p* = 0.5337). The lack of significance of storage time was influenced by the lack of variation for the Spinosad^TM^ treatment, which was 100% for all storage weeks. However, we still analyzed the data for the effect of the storage week on each individual insecticide treatment and the effect of the treatment for each individual storage week (Table 2).

The Sensat ^TM^ treatment was the most effective with 100% adult mortality at every time interval (Table 2). The Gravista^®^ treatment also had a significant reduction in adult mortality compared to the control, ranging 67–95% across all weeks of storage. In contrast, the EverGreen^®^ and Diacon^®^ IGR treatments often did not differ from the control (Table 2). Among all the treatments, adult mortality on EverGreen^®^ and Diacon^®^ IGR-treated sorghum was lower compared to Gravista^®^ and Sensat^TM^ across all storage weeks.

#### 3.2.2. *Sitophilus oryzae*

The main effects of insecticide treatment (*F* = 78.60; df = 4595; *p* < 0.0001), storage time (*F* = 15.11; df = 7592; *p* < 0.0001) and the interaction of treatment × storage time (*F* = 20.31; df = 7560; *p* < 0.0001) were significant for *S. oryzae* adult mortality. Therefore, the data on adult mortality was further analyzed for the effect of the storage week on each individual insecticide treatment and the effect of the insecticide treatment for each individual storage week, all of which were found to be significant at *p* < 0.05 (Table 3).

Similar to the results observed for *R. dominica*, the insecticide Sensat^TM^ had the highest mortality among all the treatments and ranged from 30 to 39% (Table 3) but did not differ across all the storage weeks. However, *S. oryzae* adult mortality was lower on Sensat^TM^-treated sorghum compared to *R. dominica* mortality. This trend was similar for Gravista^®^, with mortalities ranging from 0 to 41%. The mortalities of *S. oryzae* adults exposed to sorghum treated with Gravista^®^ were also higher than the control, with the exception of weeks 8 and 16. Adult mortality for Diacon^®^ IGR and EverGreen^®^ treated sorghum remained low throughout the storage period, ranging from 1.3 to 21.6% and 0 to 41.8% respectively (Table 3). Both treatments had a significant increase in adult mortality at week 20, with EverGreen^®^ reaching 42%, and Diacon^®^ IGR reaching 22%; however this was the only week that was different from the control.

### 3.3. Effect on Adult Progeny

#### 3.3.1. *Rhyzopertha dominica*

The number of *R. dominica* adult progeny was assessed eight weeks after the adults were removed from the sorghum sub-samples. All the main effects of storage time (*F* = 7.78 df = 7592, *p* < 0.0001) and treatment (*F* = 178.02 df = 4595, *p* < 0.0001) and their interaction (*F* = 82.84, df = 39,560, *p* < 0.0001) were statistically significant for the number of *R. dominica* adult progeny. Since both main effects were significant, we further analyzed for the effect of storage time on each individual insecticide treatment and the effect of the treatments for each individual storage time; all were found to be significant at *p* < 0.05 (Table 4).

With the two exceptions of EverGreen^®^ at 12 and 28 weeks, all insecticidal treatments had significantly fewer adult progeny compared to the control (Table 4). The Diacon^®^ IGR and EverGreen^®^ treatments had the lowest effects on adult mortality over the 28-week period; however both treatments reduced adult progeny compared to the control. During the course of this experiment, there was also a general decline in adult progeny observed in the control bottles. The mean adult progeny was reduced from 93 individuals at week 0 to only one adult at week 28.

#### 3.3.2. *Sitophilus oryzae*

All of the main effects of storage time (*F* = 58.63, df = 7587, *p* < 0.0001) and treatment (*F* = 14.4, df = 39,555, *p* < 0.0129), and the interaction of storage time × treatment (*F* = 14.4, df = 39,555, *p* < 0.0001) were statistically significant for the number of *S. oryzae* adult progeny. Since both main effects were significant, we further analyzed for the effect of storage time on each individual insecticide treatment and the effect of the treatments among each individual storage time (Table 5). When comparing statistical significances within storage time, only week 20 (F = 14.00, df = 4, 65, *p* < 0.0001), week 24 (F = 2.73, df = 4, 70, *p* = 0.0357), and week 28 (F = 2.65, df = 4, 70, *p* = 0.0400) were found to be significantly different.

Similar to the results observed for *R. dominica*, both the type of insecticide treatment and storage time were significant for the number of *S. oryzae* progeny that emerged. In contrast to *R. dominica* trials, none of the insecticide treatments fully suppressed progeny emergence of *S. oryzae* during the course of this experiment. While the number of *S. oryzae* progeny on Sensat^TM^-treated sorghum was not as effective compared to the number of adult *R. dominica* progeny (Table 4), Sensat^TM^ still had the fewest progeny compared to the other treatments, with a range of 9–64 individuals among all the storage weeks. All of the other insecticide treatments had progeny numbers that ranged from 6 to 84 individuals for Diacon^®^ IGR, to 8 to 104 for EverGreen^®^ and 6 to 114 for Gravista^®^. In general, adult progeny numbers decreased over time for all the treatments (Table 5). Similar to what was observed for *R. dominica* in the control bottles, the number of *S. oryzae* adult progeny observed was higher at the beginning of the experiment (week 0) and gradually declined to <9 individuals by week 28 (Table 5).

### 3.4. Grain Damage

#### 3.4.1. *Rhyzopertha dominica*

The percentage of damaged kernels of sorghum caused by *R. dominica* adults was evaluated eight weeks after the adults were removed from the sorghum sub-samples and the adult progeny were removed. All of the main effects of the storage week (*F* = 4.26, df = 7592, *p* = 0.0001), treatment (*F* = 140.94, df = 4595, *p* < 0.0001), and their interactions (*F* = 26.79, df = 39,560, *p* < 0.001) were statistically significant for the amount of frass (g) produced by *R. dominica*. Furthermore, all of the main effects of the storage week (*F* = 16.82, df = 7592, *p* < 0.0001), treatment (*F* = 190.64, df = 4595, *p* < 0.0001), and their interactions (*F* = 103.83, df = 39,560, *p* < 0.0001) were also found to be significant for the percentage of insect-damaged kernels caused by *R. dominica* adults. Since both main effects were significant, we further analyzed for the effect of the storage week on each individual insecticide treatment and the effect of the treatments for each individual storage week (Table 6 and Table 7).

Compared to the control, which had 0.16–0.99 g of frass during the course of this experiment, Diacon^®^ IGR, Gravista^®^, and Sensat^TM^ were most effective in limiting the amount of frass produced across all trial weeks. Gravista^®^ and Sensat^TM^ specifically limited the amount of frass produced to only 0.00–0.04 and 0.00–0.06 g of frass, respectively, whereas Diacon^®^ IGR limited frass production to 0.01–0.14 g of frass. While EverGreen^®^ was relatively effective when compared to the control group, the amount of frass produced by *R. dominica* was much higher than the other treatments, with 0.17–0.51 g of frass produced (Table 6).

Regarding the percentage of IDKs, all treatments were effective in reducing the amount of damage caused by *R. dominica*. Compared to the control, which had an average of 2.17–26.68% IDKs, Gravista^®^ and Sensat^TM^ were the most effective, limiting the percentage of IDKs to <1.02 and <4.11%, respectively. While also effective when compared to the control, Diacon^®^ IGR limited the percentage of IDKs <2.69%. Lastly, while again effective when compared to the control group, EverGreen^®^ was the least effective out of the treatments, limiting the percentage of IDKs to 0.77–8.54% (Table 7). Similar to the trends for progeny production of *R. dominica* during the 28-week course of this experiment, there was a noticeable decline in frass and IDKs over the storage periods.

#### 3.4.2. *Sitophilus oryzae*

All main effects of storage time (*F* = 15.11, df = 7592, *p* < 0.0001), treatment (*F* = 78.6, df = 4595, *p* < 0.0001), and their interactions (*F* = 20.31, df = 39,560, *p* < 0.0001) were statistically significant for the amount of frass (g) produced by *S. oryzae*. Furthermore, all of the main effects of storage time (*F* = 58.63, df = 7587, *p* < 0.0001), treatment (*F* = 3.18, df = 4590, *p* = 0.0134), and their interactions (*F* = 3,18, df = 39,555, *p* < 0.0001) were also found to be statistically significant for the percentage of IDKs caused by *S. oryzae* adults. Since both main effects were significant, we further analyzed for the effect of storage time on each individual insecticide treatment and the effect of the treatments among each individual storage time (Table 8 and Table 9). When comparing treatments among individual storge time periods, only week 4 (*F* = 0.31, df = 4, 70, *p* = 0.8717), week 16 (*F* = 2.29, df = 4, 70, *p* = 0.678), and week 28 (*F* = 2.35, df = 4, 70, *p* = 0.0626) did not differ from the control (Table 8), whereas only week 12 (*F* = 1.03, df = 4, 70, *p* = 0.3989) was found to not be significant for the percentage of IDKs (Table 9).

When compared to the control, the insecticide treatments were less effective at limiting the amount of frass produced by *S. oryzae* compared to *R. dominica*. This is especially true in the case of Diacon^®^ IGR which had an average of 0.07–0.95 g of frass produced, although the 0.95 g of frass resulted from adults placed on samples 24 h after initial treatment (week 0), which was an outlier compared to other time points. Regarding the other insecticide treatments EverGreen^®^ had an average of 0.09–0.39 g of frass produced, Gravista^®^ had 0.04–0.41, and Sensat^TM^ had 0.04–0.39 g. In general, Gravista^®^ was the most effective and consistent overall at limiting frass production.

Similar to the trends in frass produced, *S. oryzae* had fewer IDKs compared to *R. dominica*, ranging from 2.34 to 18.95% on the control sorghum throughout the 28 weeks (Table 9). Additionally, the effect of the insecticide treatments on IDKs were less evident against *S. oryzae* compared to *R. dominica*. We observed fluctuations in the percentage of IDKs for all treatments with intermittent increases in IDK among the storage times. Out of all the insecticide treatments, Gravista^®^ and Sensat^TM^ resulted in the highest suppression of IDKs, with 0.72–9.80 and 1.64–7.88% IDKs on average across all storage times. However, all insecticide-treated sorghum had fewer IDKs when compared to the control, excluding Evergreen^®^ at week 12 and Sensat^TM^ at week 20.

Similar to adult progeny production and IDKs, there was also a decline in frass production in the control group over time. This trend was also observed in the insecticide treatments, although frass production was more variable over time in these treatments. A similar trend was observed in IDKs, as a general decline can be seen among treatment groups along with the control (Table 9).

## 4. Discussion

*Rhyzopertha dominica* and *S. oryzae* are two key primary pests of bulk-stored grains worldwide. As internal feeders, both species complete their development within individual kernels, consuming the germ and endosperm before emerging as adults. Adult *R. dominica* deposit eggs outside the grain kernel and the newly hatched larvae bore into the kernels [13,14,15]. In contrast, *S. oryzae* females use their rostrum to bore directly into a kernel, oviposit an egg, and fill the cavity with a gelatinous material to protect the egg [15]. The larvae will develop entirely inside the kernel and emerge as an adult. This difference in oviposition behavior may partially explain why the overall number of adult *S. oryzae* progeny was higher compared to *R. dominica*, with regard to insecticides incorporating IGRs.

Throughout the study, we observed a variation in efficacy among the weeks of storage and insecticides. One factor that would contribute to the effectiveness of the grain protectants was reduction in moisture content of the sorghum over the 28-week storage period. Grain quality is constantly changing during storage, and high temperatures and reduced relative humidity increase the rate of grain drying during storage. The effect of the reduced grain moisture content is most evident in control (untreated) sorghum as the number of adult progeny, frass, and IDKs over the 28-week storage period was reduced for both species. This is likely due to the fact both species are dependent on adequate grain moisture [19], with *R. dominica’s* optimal conditions being 12–14% grain moisture in wheat [19], and *S. oryzae*’s optimal moisture content being 14–16% [20,21]. As the sorghum lost moisture during our experiment, it is likely that reduced moisture impaired egg viability, slowed development, and increased immature-stage mortality. Additionally, the nature of applying grain protectants has an innate variability during the application. The product is generally applied to a moving stream of grain and the movement of the grain during loading is expected to help move and coat the individual kernels of grain. In our study, we attempted to mimic the liquid application of a grain protectant during the top loading of grain into a silo, by applying the insecticide as the grain was poured into an empty bin and gently shaken to elicit an even coating of the individual kernels. This could impact the consistency in adult mortality and progeny among our two species tested.

The insecticide Diacon^®^ IGR contains the active ingredient methoprene, an insect IGR and juvenile hormone analog. Methoprene acts by disrupting the development of the juvenile stage of insects, inhibiting them from successfully maturing into adults after encountering the treated grain either through contact or ingestion [4]. Because methoprene primarily targets immature developmental stages, the low adult mortality observed for both species in this study was expected. However, previous research has demonstrated methoprene can have sublethal effects on adult insects [22,23]. It has been shown that *R. dominica* adults exposed to rough rice treated with 1 ppm methoprene have significantly reduced adult fecundity, with females laying 12.5 eggs/female on the methoprene-treated rice compared to 52.1 eggs/female in untreated controls [23]. In our study, adult mortality was <14% for both species; however we observed significant reduction in *R. dominica* progeny but little to no reduction in *S. oryzae* progeny. This stark difference in progeny can be associated with biological differences in oviposition between the species, as *S. oryzae* larvae would not come into contact with treated grain surfaces like the larvae of *R. dominica.* Furthermore, it was found that 1.25 ppm and 2.5 ppm (mg/kg) applications of methoprene on wheat, brown rice, and rough rice completely suppressed the progeny of *R. dominica* adults for as long as 24 weeks post-treatment [24], mirroring the results we obtained from *R. dominica* on sorghum treated with methoprene.

Gravista^®^, a formulation containing methoprene, deltamethrin, and piperonyl butoxide, demonstrated strong efficacy in suppressing *R. dominica* infestations. Adult mortality was greater than the control, thus resulting in a reduction in adult progeny and subsequent grain damage. In contrast, *S. oryzae* exhibited greater tolerance to Gravista^®^ (adult mortality < 40%), and some reduction in progeny compared to the control at times, where at other times the progeny numbers came close to or exceeded the control group. Other studies have shown increases in adult mortality in response to deltamethrin-treated grain or surfaces, which can result in reduced progeny either through the direct elimination of adults or their subsequent larval mortality [25,26]. However, some studies have shown *Sitophilus* spp. to be more tolerant of methoprene treatments when compared to other insect species tested such as *R. dominica* [25,26]. On wheat treated with 1.0 ppm deltamethrin EC alone, *S. oryzae* produced only 26 adult progeny, but when combined with methoprene the number of adult progeny was reduced to four adult *S. oryzae,* compared to 154 adult progeny in the control (untreated) wheat [26]. However, the performance of *S. oryzae* varied across different commodities [26] when exposed to deltamethrin and that greater control was achieved when deltamethrin was combined with methoprene. In our study, however, we did not achieve consistent mortality or progeny reduction with Gravista^®^. Our study suggests that the formulated insecticide Gravista^®^ may behave differently when applied to sorghum compared to wheat, rice, or maize.

EverGreen^®^, which contains natural pyrethrums, had a much lower rate of adult mortality on *R. dominica* (<25%) and *S. oryzae* (<8%) compared to the other insecticides tested. Furthermore, progeny was reduced in studies with *R. dominica*, but *S. oryzae* progeny seemed rather unaffected. The low adult mortality results of insects exposed to this insecticide when compared to other treatments such as Gravista^®^ were somewhat expected since pyrethroids, such as deltamethrin, were specifically synthesized to be more stable and toxic than the natural pyrethrums [27]. Despite this low performance when compared to other treatments, however, sorghum treated with EverGreen^®^ consistently had higher rates of adult mortality and lower rates of progeny, frass, and IDKs when compared to the control.

Sensat^TM^, which contains the a.i. spinosad, achieved complete mortality (100%) on adult *R. dominica* and subsequently no adult progeny were observed. *S. oryzae* was less susceptible to Sensat^TM^ (adult mortality < 39%), although progeny numbers for this species were lower than the control at times. Our results are similar to previous studies on wheat treated with 0.1 mg/kg of spinosad, which resulted in 96–100% adult mortality and a 94–99% progeny reduction of *R. dominica* over a nine-month residual study [28]. Additionally, previous studies on spinosad application rates of 0.5, 1.0, and 2.0 mg/kg on high vitreous wheat against three species of *Sitophilus* resulted in 34, 76, and 95% mortality, respectively, but on low vitreous wheat adult mortalities were 14, 42, and 86%, respectively [29]. This study, and ours, suggest that spinosad can behave differently on different commodities [30,31].

The varied responses of *R. dominica* and *S. oryzae* across treatments reinforce the fact that no single protectant is universally effective, and that pest-specific biology and behavior must be considered in post-harvest control strategies. Thus, it is imperative to develop an effective monitoring program to identify the species of concern [32]. While some products demonstrated excellent efficacy against one species, they were notably less effective against the other, highlighting the danger of extrapolating efficacy data from other grains or pest species to sorghum without direct evaluation. Importantly, this study reinforces the necessity of commodity- and species-specific testing under realistic storage conditions. Such research is vital not only to identify effective insecticide formulations for sorghum, but also to understand their limitations in practical use scenarios. Moreover, future work should extend beyond stand-alone treatments to consider how grain protectants integrate with other IPM components, such as grain aeration and fumigation, to build a more comprehensive IPM program for sorghum.

## Figures and Tables

**Table 1:** Mean (±SE) percent moisture content of sorghum held in 18.9 L plastic buckets in an empty grain bin over a 28-week period. Means followed by different uppercase letters indicate statistically significant differences among the weeks of storage (*p* < 0.05, Proc GLM, LS Means with Tukey’s HSD test).

Weeks of Storage	% Moisture Content
0	13.30 ± 0.21 A
4	12.83 ± 0.12 A
8	12.03 ± 0.15 B
12	11.40 ± 0.24 CD
16	11.57 ± 0.14 BC
20	10.87 ± 0.10 DE
24	10.50 ± 0.05 E
28	9.93 ± 0.08 F

**Table 2:** Mean (±SE) percent mortality of adult *Rhyzopertha dominica* seven days post-exposure to sorghum treated with water (control), Diacon^®^ IGR, EverGreen^®^, or Gravista^®^, or Sensat^TM^ and held for each of eight storage periods. Means within rows for each storage week followed by different lowercase letters are significant, and means within columns for each insecticide followed by different capital letters are significant (*p* < 0.05, Proc GLM, LS Means with Tukey’s HSD test).

Weeks of Storage	Control	Diacon^®^ IGR	EverGreen^®^	Gravista^®^	Sensat^TM^
0	8.67 ± 2.15 cAB	4.67 ± 1.92 cdABC	2.00 ± 1.17 dD	89.33 ± 3.58 bA	100.00 ± 0.00 aA
4	5.33 ± 1.65 cBC	5.33 ± 1.65 cAB	3.41 ± 1.29 cD	90.00 ± 6.62 bA	100.00 ± 0.00 aA
8	3.83 ± 1.59 cCD	7.33 ± 2.28 cAB	24.07 ± 5.04 bAB	92.67 ± 5.39 aA	100.00 ± 0.00 aA
12	8.83 ± 2.75 cABC	4.00 ± 2.35 dBC	12.81 ± 1.87 cBC	94.67 ± 2.15 bA	100.00 ± 0.00 aA
16	4.15 ± 1.36 cBCD	6.00 ± 1.63 cAB	25.27 ± 6.10 bA	94.67 ± 2.56 aA	100.00 ± 0.00 aA
20	8.16 ± 3.42 cBC	11.82 ± 3.66 cA	12.00 ± 2.99 cC	80.00 ± 10.69 bAB	100.00 ± 0.00 aA
24	0.67 ± 0.67 cD	0.00 ± 0.00 cC	2.00 ± 1.07 cD	72.76 ± 4.90 bBC	100.00 ± 0.00 aA
28	14.09 ± 2.67 cA	5.65 ± 2.46 dABC	23.26 ± 4.61 cAB	66.67 ± 6.45 bC	100.00 ± 0.00 aA

**Table 3:** Mean (±SE) percent mortality of adult *Sitophilus oryzae* seven days post-exposure to sorghum treated with water (control), Diacon^®^ IGR, EverGreen^®^, Gravista^®^, or Sensat^TM^ and held for each of eight storage periods. Means within rows for each storage week followed by different lowercase letters are significant, and means within columns for each insecticide followed by different capital letters are significant (*p* < 0.05, Proc GLM, LS Means with Tukey’s HSD test).

Weeks of Storage	Control	Diacon^®^ IGR	EverGreen^®^	Gravista^®^	Sensat^TM^
0	1.33 ± 0.91 cBC	1.33 ± 0.91 cC	0.00 ± 0.00 cD	14.00 ± 3.49 bC	36.67 ± 5.58 aA
4	3.33 ± 1.26 cBC	3.33 ± 1.59 cBC	4.48 ± 1.56 bcBC	13.10 ± 4.04 bC	38.82 ± 5.65 aA
8	0.67 ± 0.67 bC	1.33 ± 0.91 bC	0.67 ± 0.67 bCD	0.00 ± 0.00 bE	30.00 ± 4.58 aA
12	4.67 ± 1.65 cB	7.94 ± 2.22 bcB	6.00 ± 2.35 bcB	12.00 ± 2.79 bC	34.00 ± 6.31 aA
16	3.33 ± 1.26 bBC	2.67 ± 1.18 bBC	4.00 ± 1.63 bBCD	3.33 ± 1.59 bDE	37.33 ± 6.05 aA
20	4.15 ± 1.36 cB	21.63 ± 5.93 bA	41.78 ± 6.11 aA	40.67 ± 6.86 aA	38.00 ± 5.45 aA
24	0.61 ± 0.61 cC	4.00 ± 1.31 bcBC	3.33 ± 1.25 bcBCD	6.67 ± 2.11 bCD	30.00 ± 4.8 aA
28	12.83 ± 2.66 bA	14.00 ± 2.14 bA	8.00 ± 2.00 bB	27.33 ± 5.30 aB	34.93 ± 3.49 aA

**Table 4:** Mean (±SE) emerged *Rhyzopertha dominica* adult progeny from sorghum treated with water (control), Diacon^®^ IGR, EverGreen^®^, Gravista^®^, or Sensat^TM^ and held for each of eight storage periods. Means within rows for each storage week followed by different lowercase letters are significant, and means within columns for each insecticide followed by different capital letters are significant (*p*< 0.05, Proc GLM, LS Means with Tukey’s HSD test).

Weeks of Storage	Control	Diacon^®^ IGR	EverGreen^®^	Gravista^®^	Sensat^TM^
0	93.33 ± 11.23 aA	0.07 ± 0.07 cC	12.80 ± 2.04 bA	0.00 ± 0.00 cC	0.00 ± 0.00 cC
4	81.87 ± 9.53 aA	0.87 ± 0.27 cAB	5.67 ± 1.39 bB	0.60 ± 0.24 cB	0.27 ± 0.12 cBC
8	72.67 ± 8.63 aA	2.73 ± 0.99 cA	5.60 ± 1.27 bB	0.53 ± 0.24 cdB	0.20 ± 0.14 dC
12	3.53 ± 0.67 aD	0.47 ± 0.17 bBC	4.27 ± 1.27 aB	0.20 ± 0.11 bBC	0.20 ± 0.14 bC
16	49.67 ± 7.01 aB	0.47 ± 0.19 cBC	0.33 ± 0.16 cD	5.93 ± 1.28 bA	0.07 ± 0.07 cC
20	29.53 ± 4.86 aBC	0.67 ± 0.23 cBC	9.67 ± 1.64 bA	0.27 ± 0.15 cBC	10.40 ± 0.79 bA
24	20.47 ± 3.50 aC	0.33 ± 0.13 cBC	2.53 ± 0.57 bBC	0.13 ± 0.09 cBC	1.00 ± 0.65 cB
28	1.27 ± 0.32 aD	0.53 ± 0.17 bcBC	0.87 ± 0.17 abCD	0.20 ± 0.20 cBC	0.13 ± 0.13 cC

**Table 5:** Mean (±SE) emerged *Sitophilus oryzae* adult progeny from sorghum treated with water (control), Diacon^®^ IGR, EverGreen^®^, Gravista^®^, or Sensat^TM^ and held for each of eight storage periods. Means within rows for each storage week followed by different lowercase letters are significant, and means within columns for each insecticide followed by different capital letters are significant (*p* < 0.05, Proc GLM, LS Means with Tukey’s HSD test).

Weeks of Storage	Control	Diacon^®^ IGR	EverGreen^®^	Gravista^®^	Sensat^TM^
0	107.07 ± 10.49 aA	83.87 ± 15.28 aA	104.13 ± 10.20 aA	114.00 ± 15.57 aA	64.33 ± 8.63 aA
4	41.00 ± 6.73 aBCD	64.20 ± 9.67 aA	48.07 ± 8.85 aBC	49.73 ± 9.46 aB	42.87 ± 6.45 aAB
8	47.60 ± 5.35 aBC	48.60 ± 6.81 aA	45.73 ± 8.15 aBC	60.73 ± 8.83 aAB	43.13 ± 8.37 aABC
12	28.67 ± 3.81 aD	23.13 ± 3.10 aB	29.53 ± 5.38 aC	25.13 ± 4.76 aCD	20.13 ± 2.50 aC
16	40.73 ± 3.63 aBC	35.20 ± 4.56 aAB	31.47 ± 5.02 aBC	37.47 ± 5.01 aBC	42.67 ± 3.54 aAB
20	53.53 ± 6.17 aB	45.80 ± 4.99 aA	58.60 ± 6.88 aB	22.53 ± 3.80 bD	13.67 ± 3.29 cD
24	34.33 ± 4.54 aCD	18.40 ± 2.53 bB	26.87 ± 3.96 abC	19.20 ± 4.23 bD	26.00 ± 3.70 abBC
28	8.47 ± 1.52 aE	6.13 ± 1.93 abC	8.33 ± 1.27 aD	3.80 ± 0.88 bE	8.80 ± 1.73 aD

**Table 6:** Mean (±SE) amount of frass (g) from *Rhyzopertha dominica* eight weeks post-exposure to sorghum treated with water (control), Diacon^®^ IGR, EverGreen^®^, Gravista^®^, or Sensat^TM^ and held for each of eight storage periods. Means within rows for each storage week followed by different lowercase letters are significant, and means within columns for each insecticide followed by different capital letters are significant (*p* < 0.05, Proc GLM, LS Means with Tukey’s HSD test).

Weeks of Storage	Control	Diacon^®^ IGR	EverGreen^®^	Gravista^®^	Sensat^TM^
0	0.54 ± 0.08 aB	0.14 ± 0.05 cA	0.31 ± 0.07 bB	0.01 ± 0.00 dBC	0.00 ± 0.00 dB
4	0.99 ± 0.11 aA	0.07 ± 0.04 cAB	0.17 ± 0.03 bB	0.02 ± 0.01 cABC	0.03 ± 0.03 cAB
8	0.25 ± 0.08 aCD	0.02 ± 0.01 bB	0.17 ± 0.03 aB	0.00 ± 0.00 bC	0.00 ± 0.00 bB
12	0.87 ± 0.12 aA	0.03 ± 0.00 cB	0.51 ± 0.09 bA	0.04 ± 0.01 cA	0.00 ± 0.00 cB
16	0.44 ± 0.07 aB	0.03 ± 0.01 cB	0.23 ± 0.03 bB	0.03 ± 0.01 cAB	0.06 ± 0.03 cA
20	0.48 ± 0.06 aB	0.01 ± 0.00 cB	0.25 ± 0.03 bB	0.00 ± 0.00 cC	0.00 ± 0.00 cB
24	0.39 ± 0.05 aBC	0.07 ± 0.04 cAB	0.18 ± 0.02 bB	0.01 ± 0.01 cBC	0.01 ± 0.01 cB
28	0.15 ± 0.06 aD	0.10 ± 0.08 abAB	0.22 ± 0.11 aB	0.00 ± 0.00 bC	0.00 ± 0.00 bB

**Table 7:** Mean (±SE) percent of insect damaged kernels (IDKs) from *Rhyzopertha dominica* eight weeks post-exposure to sorghum treated with water (control), Diacon^®^ IGR, EverGreen^®^, Gravista^®^, or Sensat^TM^ and held for each of eight storage periods. Means within rows for each storage week followed by different lowercase letters are significant, and means within columns for each insecticide followed by different capital letters are significant (*p* < 0.05, Proc GLM, LS Means with Tukey’s HSD test).

Weeks of Storage	Control	Diacon^®^ IGR	EverGreen^®^	Gravista^®^	Sensat^TM^
0	26.68 ± 2.35 aA	2.52 ± 0.29 cAB	7.21 ± 0.84 bA	0.65 ± 0.09 dAB	0.94 ± 0.24 dB
4	28.21 ± 1.76 aA	2.69 ± 0.20 cA	6.36 ± 0.58 bA	1.01 ± 0.08 dA	0.99 ± 0.15 dB
8	18.84 ± 1.88 aB	2.53 ± 0.29 cAB	7.89 ± 0.65 bA	0.97 ± 0.13 dA	0.79 ± 0.14 dB
12	14.11 ± 1.39 aC	1.91 ± 0.24 cB	8.54 ± 1.08 bA	1.02 ± 0.17 cdA	0.80 ± 0.20 dB
16	10.14 ± 0.88 aC	2.17 ± 0.23 cAB	0.68 ± 0.11 dC	0.73 ± 0.12 dA	4.11 ± 0.54 bA
20	5.85 ± 0.70 aD	1.07 ± 0.16 cC	3.60 ± 0.28 bB	0.52 ± 0.21 dBC	0.11 ± 0.08 eC
24	5.04 ± 0.36 aD	1.87 ± 0.17 cB	3.26 ± 0.40 bB	0.22 ± 0.06 dCD	0.22 ± 0.08 dC
28	2.17 ± 0.35 aE	0.42 ± 0.08 bD	0.77 ± 0.16 bC	0.10 ± 0.06 cD	0.02 ± 0.02 cC

**Table 8:** Mean (±SE) amount of frass (g) from *Sitophilus oryzae* eight weeks post-exposure to sorghum treated with water (control), Diacon^®^ IGR, EverGreen^®^, Gravista^®^, or Sensat^TM^ and held for each of eight storage times. Means within rows for each storage week followed by different lowercase letters are significant, and means within columns for each insecticide followed by different capital letters are significant (*p* < 0.05, Proc GLM, LS Means with Tukey’s HSD test).

Weeks of Storage	Control	Diacon^®^ IGR	EverGreen^®^	Gravista^®^	Sensat^TM^
0	0.42 ± 0.07 bAB	0.95 ± 0.25 aA	0.26 ± 0.02 bcA	0.14 ± 0.02 cBCD	0.26 ± 0.04 bcAB
4	0.29 ± 0.09 aBA	0.27 ± 0.04 aB	0.37 ± 0.08 aA	0.41 ± 0.15 aA	0.36 ± 0.09 aA
8	0.45 ± 0.07 aA	0.24 ± 0.04 bcBC	0.39 ± 0.09 abA	0.16 ± 0.02 cB	0.39 ± 0.06 abA
12	0.10 ± 0.02 abD	0.07 ± 0.01 bC	0.09 ± 0.02 bB	0.05 ± 0.01 bCD	0.18 ± 0.06 aBC
16	0.14 ± 0.01 aCD	0.10 ± 0.01 aC	0.10 ± 0.02 aB	0.15 ± 0.01 aBC	0.12 ± 0.02 aCD
20	0.27 ± 0.03 aBC	0.23 ± 0.03 aBC	0.26 ± 0.04 aA	0.12 ± 0.02 bBCD	0.05 ± 0.01 cD
24	0.30 ± 0.12 aBC	0.09 ± 0.03 bC	0.10 ± 0.02 bB	0.11 ± 0.04 bBCD	0.08 ± 0.01 bCD
28	0.05 ± 0.01 aD	0.09 ± 0.02 aC	0.14 ± 0.06 aB	0.04 ± 0.01 aD	0.04 ± 0.01 aD

**Table 9:** Mean (±SE) percent of insect damaged kernels (IDKs) by *Sitophilus oryzae* eight weeks post-exposure to sorghum treated with water (control), Diacon^®^ IGR, EverGreen^®^, Gravista^®^, or Sensat^TM^ and held for each of eight storage periods. Means within rows for each storage week followed by different lowercase letters are significant, and means within columns for each insecticide followed by different capital letters are significant (*p* < 0.05, Proc GLM, LS Means with Tukey’s HSD test).

Weeks of Storage	Control	Diacon^®^ IGR	EverGreen^®^	Gravista^®^	Sensat^TM^
0	18.95 ± 1.96 aA	13.29 ± 1.97 bA	9.95 ± 0.97 bcA	8.06 ± 0.89 cAB	7.88 ± 1.14 cA
4	9.05 ± 1.32 aBC	8.59 ± 1.04 aB	6.49 ± 1.10 abB	5.36 ± 0.76 bCD	7.28 ± 1.01 abAB
8	10.71 ± 1.21 aB	8.52 ± 1.18 aB	9.25 ± 1.19 aA	9.80 ± 1.20 aA	7.21 ± 1.03 aAB
12	5.50 ± 0.60 D	4.60 ± 0.52 DC	4.72 ± 0.83 B	3.96 ± 0.40 D	5.85 ± 0.93 AB
16	9.97 ± 1.15 aBC	7.60 ± 1.73 abBC	6.41 ± 0.94 bB	7.10 ± 0.69 abBC	5.26 ± 0.67 bB
20	9.09 ± 0.87 aBC	8.30 ± 0.84 aB	10.41 ± 1.21 aA	5.82 ± 0.80 bCD	2.67 ± 0.44 cC
24	7.09 ± 0.80 aDC	2.63 ± 0.34 dDE	4.57 ± 0.51 bcB	4.12 ± 0.48 cD	5.99 ± 0.48 abAB
28	2.34 ± 0.28 aE	1.92 ± 0.20 abE	1.37 ± 0.20 bC	0.72 ± 0.14 cE	1.64 ± 0.23 bC

## Data Availability

Data are available on Ag Data Commons through the National Agricultural Library at 10.15482/USDA.ADC/30132415.
